# Direct Regulation of Pitx3 Expression by Nurr1 in Culture and in Developing Mouse Midbrain

**DOI:** 10.1371/journal.pone.0030661

**Published:** 2012-02-17

**Authors:** Floriana Volpicelli, Roberto De Gregorio, Salvatore Pulcrano, Carla Perrone-Capano, Umberto di Porzio, Gian Carlo Bellenchi

**Affiliations:** 1 Institute of Genetics and Biophysics “Adriano Buzzati-Traverso”, Naples Italy; 2 University of Naples “Federico II” Department of Biological Sciences, Naples, Italy; INSERM, UMR-S747, France

## Abstract

Due to their correlation with major human neurological diseases, dopaminergic neurons are some of the most studied neuronal subtypes. Mesencephalic dopaminergic (mDA) differentiation requires the activation of a cascade of transcription factors, among which play a crucial role the nuclear receptor *Nurr1* and the paired-like homeodomain 3, *Pitx3*. During development the expression of Nurr1 precedes that of Pitx3 and those of typical dopaminergic markers such as *tyrosine hydroxylase* (*TH*) and *dopamine Transporter* (DAT) that are directly regulated by Nurr1. Interestingly we have previously demonstrated that Nurr1 RNA silencing reduced *Pitx3* transcripts, leading to the hypothesis that Nurr1 may control *Pitx3* expression.

Here we show that Nurr1 overexpression up-regulates that of *Pitx3* in a dose-dependent manner by binding to a non-canonical NBRE consensus sequence, located at the 5′ site of the gene. Interestingly, this sequence shows the same effect as the canonical one in promoting gene translation, and its deletion abolishes the ability of Nurr1 to sustain reporter gene expression. Moreover, we show that there is a direct interaction between Nurr1 and the *Pitx3* gene promoter in dopaminergic cell cultures and midbrain embryonic tissue. Altogether, our results suggest that the regulation of *Pitx3* by Nurr1 may be an essential event controlling the development and function of mDA neurons.

## Introduction

Mesencephalic dopaminergic (mDA) neurons play a key role in the motor, reward and emotional behavior of mammals. They are located in the ventral midbrain forming three distinct nuclei, the *substantia nigra* (SN), the ventral tegmental area, and the retrorubral red nucleus; together they constitute only about 1–5% of the midbrain cell population [1]. The high incidence of their degeneration in older people (Parkinson's disease, PD), as well as their involvement in widespread neuropsychiatric diseases (schizophrenia or attention deficit hyperactive disorder, ADHD), have prompted great efforts toward understanding the molecular mechanisms underlying their specification, differentiation and maintenance.

During embryonic development in rodents, the activation of specific genes encoding for transcription factors (TFs) establishes a molecular code essential for the proper maturation and differentiation of terminal mDA. These include the member 2 nuclear receptor subfamily 4 group A (*Nr4a2* or *Nurr1*) whose expression has been detected already at E10.5 in the mouse ventral mesencephalon [2], [3], and the paired-like homeodomain transcription factor 3 (*Pitx3*) [4], [5], [6]. Pitx3 is also expressed early during mDA differentiation, starting at E11 and before the onset of the typical DA markers. Both TFs persist throughout life, albeit at lower levels than during development.

Nurr1, differently from Pitx3, whose expression is restricted to DA neurons [6], [7], is also present in other regions of the mammalian brain, as well as outside the nervous system [8]. Indeed its expression has been detected in non-dopaminergic areas such as cerebral cortex and hippocampus [9], [10] and in microglial cells [11], where it is involved in the modulation of the inflammatory response. Thus these findings suggest that it could exert a wider transcriptional control than that described for the mDA system.

The key role of Nurr1 during mDA phenotype development and survival has been highlighted by conventional knock-out mouse model and by conditional deletion [12]. In both cases it has been shown that the deletion of Nurr1 determines the progressive loss of mDA neurons, suggesting that its presence is essential for maintenance of the dopaminergic specification throughout the entire DA neuron lifespan. On the other hand, the alteration of Pitx3 in the naturally occurring mouse mutant aphakia shows a selective depletion of mDA neurons in the *substantia nigra* [4], [6].

The mechanism of action of Nurr1 has been dissected *in vitro* and a number of its target genes in mDA neurons have been identified, including *tyrosine hydroxylase* (*TH*), *vesicular monoamine transporter 2* (*Vmat2)*, *dopamine transporter* (*DAT*), *Neuropilin*, and *brain derived neurotrophic factor* (*Bdnf*) [13]–[17]. Some of these genes, such as *Vmat2*, *TH* and *DAT*, are targets of Pitx3 as well [18]. The involvement of both TFs in the regulation of crucial mDA genes suggests that these proteins might cooperate and participate in several processes during mDA neuron development, but their hierarchical relationship remains unknown.

We have previously reported, by using midbrain cultures enriched in mDA neurons, that silencing *Nurr1* determines a significant decrease of *Pitx3* expression similar to that observed for other gene targets of Nurr1 such as *Bdnf* and *TH* [17]. This finding has prompted us to investigate if the expression level of Pitx3 may depend on Nurr1. Here we show that Nurr1 directly regulates *Pitx3* expression by binding to a specific Nurr1-binding region located in the *Pitx3* gene promoter.

## Results

### Overexpression of Nurr1 up-regulates *Pitx3* mRNA and protein

**Figure 1:**
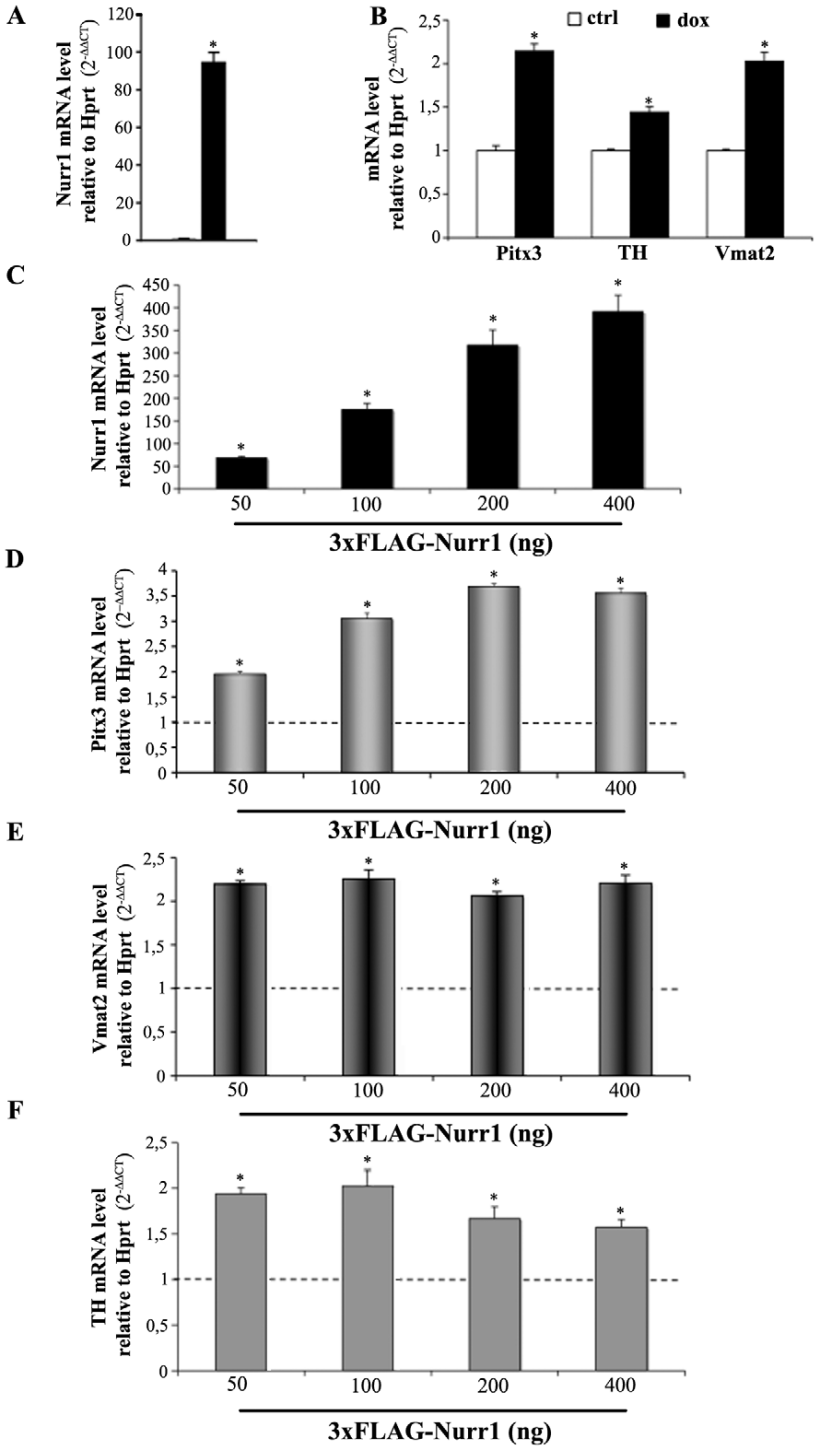
Overexpression of Nurr1 up-regulates *Pitx3, TH and Vmat2* mRNAs. Transcriptional analysis performed by real time PCR of *Nurr1*, *Pitx3*, *TH* and *Vmat2* in MN9D cells treated with 3 µg/ml of doxycycline (dox, A, B) or with different concentrations (ng) of 3×FLAG-Nurr1 plasmid (C, D, E, F). The diagrams (C–F) show the mRNA levels of *Nurr1*, *Pitx3*, *TH* and *Vmat2* over an empty vector as control (dotted lines, mean±SE). Expression levels are presented as the relative number of copies compared with the Hprt transcript using the comparative threshold cycle (CT) method (2^−ΔΔ*CT*^). Asterisks (*) represent p≤0.01 when compared to relative controls (ANOVA, Scheffè F-test).

To investigate whether or not Nurr1 affects *Pitx3* expression we used the MN9D-Nurr1^Tet-On^ cell line, which expresses Nurr1 under a tetracycline-inducible promoter [13]. MN9D cells have already been used as the cellular model system to study the development and maturation of mDA neurons. They are able to synthesize and release dopamine, and after Nurr1 overexpression acquire a more pronounced dopaminergic phenotype by boosting the expression of the dopaminergic-related markers such as *TH* and *Vmat2* [13]. Following Nurr1 hyper-expression by doxycycline treatment (Figure 1A) we observed a two-fold enrichment in *Pitx3* mRNA, when compared to control cultures (Figure 1B). To confirm the role of Nurr1 as an inducer of the DA phenotypes we observed a parallel up-regulation of the levels of *TH* and *Vmat2* mRNA, in agreement with previous data (Figure 1B) [13].

Since the expression of *Pitx3* was only slightly increased by dox-mediated overexpression of Nurr1 we replicated similar experiments by transfecting the parental MN9D cell line with a 3×FLAG-Nurr1 plasmid, which gave a more sustained *Nurr1* expression (Figure 1C). As shown in Figure 1D the up-regulation of *Pitx3* was proportional to the amount of the Nurr1-expressing plasmid when compared to cells transfected with an empty vector. Under these conditions we observed an increase of *Pitx3* mRNA up to 3.5 times above the control. Similarly *Vmat2* and *TH* mRNAs were also up regulated and their higher level of expression was achieved at a lower concentration of Nurr1 plasmid, reaching a plateau at about 50 ng/well. (Figure 1E–F, respectively).

**Figure 2:**
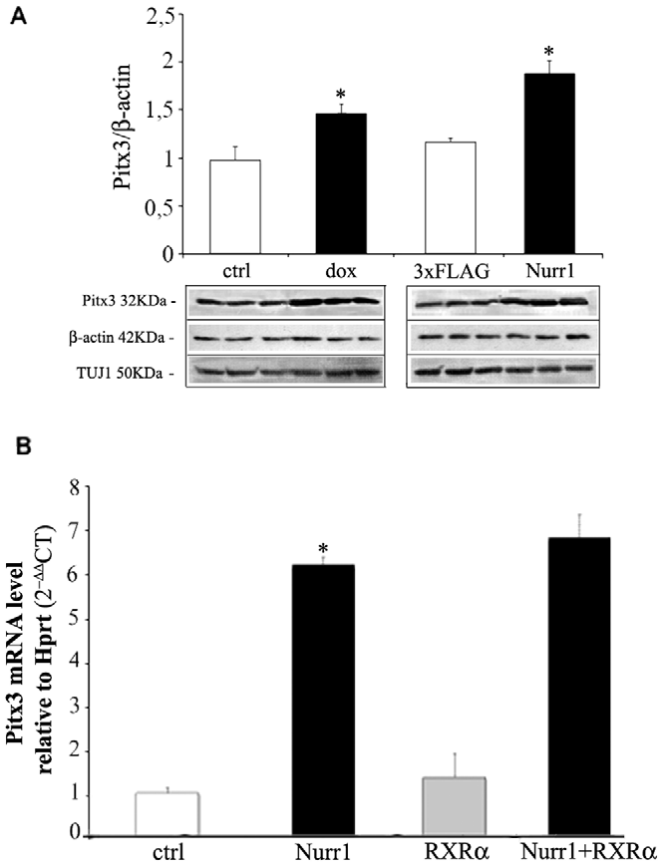
Pitx3 up-regulation depends of Nurr1 overexpression. (A) Pitx3 protein expression in MN9D treated or not (ctrl) with 3 µg/ml of doxycycline (dox) and transfected with the 3×FLAG-Nurr1 expressing vector (Nurr1) or with an empty vector (3×FLAG). The diagram shows the relative quantitation (mean±SE) of Pitx3 protein compared to that of ß-actin. The relative abundance of the Neuronal Class III β-Tubulin (TUJ1) is shown as an additional loading control. Data are expressed as ratio Pitx3/ß-actin. Inserts show a representative western blot of Pitx3, ß-actin and TUJ1. (B) *Pitx3* mRNA level in MN9D cells transfected with: 3×FLAG-Nurr1 (Nurr1), psg5-RXR (RXRα), or both constructs (Nurr1+RXRα). The diagram shows *Pitx3* mRNA level (mean±SE) relative to Hprt (2^−ΔΔ*CT*^). Asterisks (*) represent p≤0.01 when compared to controls (ANOVA, Scheffè F-test).

An increase of Pitx3, up to 50%, was also observed at the protein level (Figure 2A) with respect to untreated samples. Such up-regulation was observed both in MN9D-Nurr1^Tet-On^ cells upon doxycycline treatment and MN9D cells after Nurr1 transfection. The relative expression of Pitx3 was assessed by quantifying proteins bands, under the various experimental conditions, and normalized to ß-actin. Similar results were obtained by using the Neuronal Class III ß-Tubulin (TUJ1) as internal control (Figure 2A).

Since Nurr1 can regulate transcription either as a monomer or as a heterodimer with retinoid X receptor (RXR) [19], we investigated whether the effects of Nurr1 on *Pitx3* expression were modified upon dimerization of Nurr1 with RXR. First we established that RXR alone was unable to increase *Pitx3* mRNA above control levels (Figure 2B); then we co-transfected Nurr1 and RXRα to assess for the existence of any synergic effect. As shown in Figure 2B, the co-expression of Nurr1 and RXRα does not modify *Pitx3* mRNA levels, thus suggesting that Nurr1 acts as either a monomer or homodimer in controlling Pitx3 translation.

### The mouse *Pitx3* promoter is responsive to Nurr1

**Figure 3:**
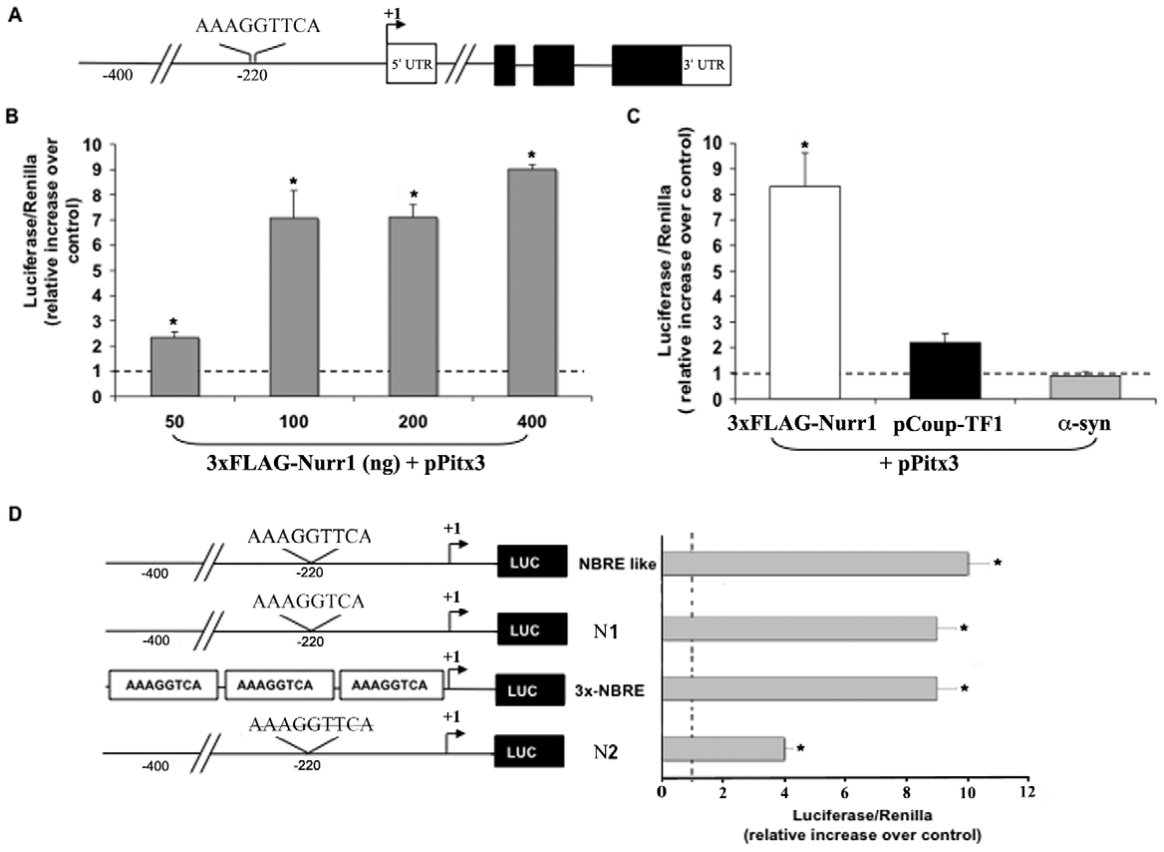
The mouse Pitx3 promoter is responsive to Nurr1. (A) Schematic representation of the mouse *Pitx3* gene indicating the position of NBRE-like binding sites in *Pitx3* promoter. (B) Luciferase assay in HeLa cells co-transfected with the *Pitx3* promoter–reporter vector and with increasing concentration of 3×FLAG-Nurr1 plasmid. (C) The *Pitx3* promoter activation is specific for Nurr1 since vectors expressing Coup-TF1 (chicken ovalbumin upstream promoter Transcription Factor 1) or alpha-synuclein (α-syn) were unable to promote luciferase expression. (D) Scheme of the promoter–reporter gene constructs used. We named: i) “NBRE-like” the promoter with the endogenous sequence; ii) “N1” the endogenous promoter deleted of the extra T to restore the canonical NBRE sequence; iii) “N2” the endogenous promoter deleted of the entire NBRE-like region. As positive control we used a reporter vector expressing three times the canonical NBRE sequence (3×-NBRE). The histogram shows similar luciferase activation when NBRE-like, N1 or 3×-NBRE constructs were used. The deletion of the entire NBRE-like region (N2) significantly reduced luciferase expression. The ratio of firefly luciferase/Renilla activity is expressed as relative increase over control (dotted lines). The results are expressed as mean ± SE; asterisks (*) represent p≤0.01 when compared to control (ANOVA, Scheffè F-test).

We then investigated if Nurr1 could control the expression of *Pitx3* at the transcriptional level. By performing *in silico* analysis of the *Pitx3* promoter region we did not find canonical NBRE sequences (AAAGGTCA), known to be essential for Nurr1 binding. Instead our analysis revealed a potential consensus motif, located 220 base pairs (bp) upstream the transcription initiation site that differs from the canonical NBRE for the insertion of a T right after the central core (AAAGGT**T**CA; Figure 3A). We renamed this sequence NBRE-like. To assess whether Nurr1 could directly interact with the *Pitx3* promoter and stimulate transcription, we cloned the 700 bp region in a reporter vector above the Pitx3 transcription initiation site, and verified that it contained the NBRE-like region by sequencing it. To assess that it could trigger transcription, we performed a luciferase assay in the human cell line HeLa. As shown in Figure 3B, Nurr1 was able to activate transcription of the *luc*-gene under the control of the *Pitx3* promoter. This activation was specific for Nurr1 since vectors expressing Coup-TF1 (chicken ovalbumin upstream promoter transcription factor 1), a member of the nuclear receptor transcription factor superfamily, or an unrelated protein such as the alpha-synuclein, were unable to promote luciferase expression (Figure 3C).

Next, to understand whether the ability of the NBRE-like consensus site to promote reporter gene expression was comparable to that of the NBRE, we restored the canonical motif by deleting the extra T (construct N1; AAAGGTTCA in AAAGGTCA). As we hypothesized, this mutation did not modify the ability of the *Pitx3* promoter to activate the reporter transcription. These data confirm that the NBRE-like region is able to bind Nurr1 and thus activate transcription of the downstream gene, with efficiency comparable to the NBRE sequence. In addition, the transcriptional stimulation triggered by the NBRE-like sequence was comparable to that obtained by using a construct carrying three repeated NBRE binding sites (3×-NBRE) (Figure 3D). Finally, to further confirm that the NBRE-like region was the only Nurr1-dependent sequence able to promote *Pitx3* gene transcription, we deleted the 20 bp containing this region (gggcctAAAGGTTCAcagct). As expected, the deletion abolished over 70% of the luciferase expression (Figure 3D).

These data confirm that the NBRE-like sequence we identified on the *Pitx3* promoter is a proper target of Nurr1.

### Nurr1 regulates *Pitx3* expression by binding to its promoter

**Figure 4:**
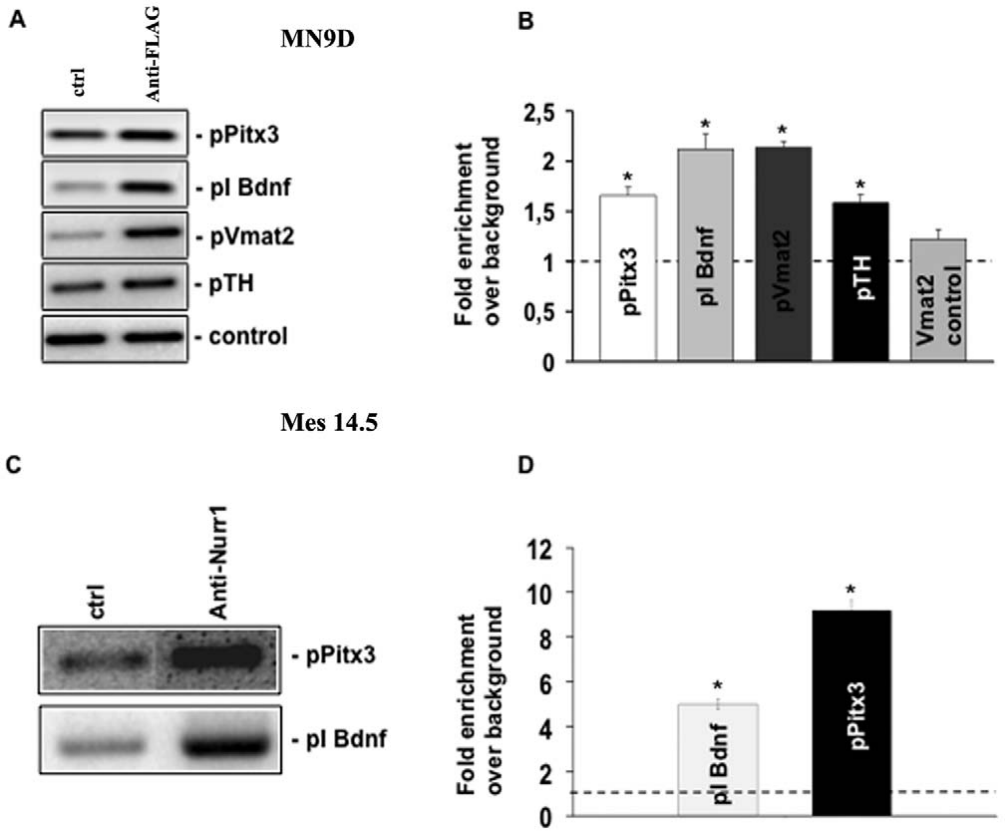
Nurr1 directly regulates *Pitx3* expression by binding to its promoter. (A) ChIP-PCR analysis performed in MN9D transfected with 3×FLAG-Nurr1 and immunoprecipitated with anti-FLAG antibody shows a significant enrichment of *Pitx3*, *Bdnf* and *Vmat2* promoter regions. No enrichment was observed when an unrelated region of the *Vmat2* promoter was used [19]. The inserts show representative PCR amplified fragments after ChIP. (B) ChIP-Real time PCR in MN9D transfected with 3×FLAG-Nurr1 and immunoprecipitated with anti-FLAG antibody. The diagram shows the fold enrichment over background (dotted line) for *Pitx3*, *Bdnf* and *Vmat2* promoter regions. (C) ChIP-PCR validation performed in E14.5 midbrain and immunoprecipitated with Nurr1 antibody shows a significant enrichment of the *Pitx3* and *Bdnf* promoter regions. A representative PCR amplified fragment is shown into the insert. (D) The diagram shows the ChIP-Real time PCR quantitation of *Bdnf* and *Pitx3* promoter region in E14.5 midbrain and immunoprecipitated with Nurr1 antibody. Results are expressed as mean ± SE of at least three independent experiments. Asterisks (*) represent p≤0.01 when compared to control (ANOVA, Scheffè F-test).

We performed a chromatin immunoprecipitation (ChIP) assay to demonstrate that Nurr1 can bind the genomic NBRE-like sequence upstream to Pitx3. Indeed by using the immunoprecipitate from MN9D cells overexpressing 3×FLAG-Nurr1, we were able to show a significant enrichment of the *Pitx3* promoter fragment containing the NBRE-like sequence, either by PCR (Figure 4A) or real time PCR (Figure 4B). We also probed this same immunoprecipitate for the presence of promoter regions of other Nurr1 target genes such as *Bdnf*, *Vmat2* and *TH*, and found that they were all enriched. As expected, no enrichment was observed when we amplified a part of Vmat2 promoter that did not contain the Nurr1 consensus sequence.

In order to confirm that Nurr1 binds to the NBRE-like sequence on the *Pitx3* also in vivo, we repeated the ChIP analysis on mouse ventral midbrain tissue during embryonic development (at E14.5). This time point was chosen since the highest Nurr1 expression takes place at this developmental stage in this region. We observed a significant enrichment of the *Pitx3* promoter fragment containing the NBRE-like sequence, even higher (about seven-fold) than that observed in cell cultures (Figure 4C–D). In addition we found a four-fold enrichment of the *Bdnf* promoter region (Figure 4C–D), as expected [17].

Altogether, these data strongly support a direct regulation of *Pitx3* expression by Nurr1.

## Discussion

During ontogeny, the midbrain regional identity requires the action of a complex transcriptional program involving among others the TFs *Lmx1a*, *Lmx1b*, *Pitx3* and *Nurr1* [20]. This last TF appears to be a major player in the control of the mDA phenotype, through the regulation of several proteins required for dopaminergic function. However, it has been shown that also Pitx3 is able to promote the expression of DA genes, suggesting a role for both TFs as players acting on the same pathways. Thus several approaches have been used, aimed at clarifying the reciprocal roles of the transcription factors *Nurr1* and *Pitx3* in promoting the differentiation of mDA neurons. By using molecular and cellular approaches, a number of common downstream target genes of both proteins have been identified, such as the vesicular transporter *Vmat2* and the plasma membrane transporter *DAT* [13], [16]. In addition Nurr1 −/− mice display the absence of TH^+^ neurons in the ventral midbrain, because their development is incomplete [21]. Indeed a few cells transiently expressed Pitx3 in this area but disappeared shortly thereafter leading to two alternative hypotheses: either *Nurr1* expression is required to maintain *Pitx3* levels, or *Pitx3* expression is independent of Nurr1 and is lost by the early death of the mDA neurons. Moreover the combined transduction of *Nurr1* and *Pitx3* promotes the maturation of ES cells into a dopaminergic phenotype [22]. These observations have prompted the idea that a hierarchical relationship could exist between Nurr1 and Pitx3, or alternatively that both transcription factors could cooperate at the protein level in controlling the dopaminergic transcription machinery.

Since in previous *Nurr1* silencing experiments we observed a reduction of *Pitx3* mRNA, we were prompted to investigate whether and how Nurr1 could modulate the expression of *Pitx3*. Here we show that Nurr1 can increase *Pitx3* transcripts and that this effect is achieved by the specific binding of Nurr1 to a non-canonical NBRE element located 220 bp upstream the transcription initiation site on the *Pitx3* promoter. By using multiple approaches involving overexpression of Nurr1 in a dopaminergic cell line, luciferase reporter assay and chromatin immunoprecipitation, in both culture and embryonic midbrain tissues, we provide evidence that Nurr1 does indeed promote *Pitx3* expression. Thus we suggest that Nurr1 controls the specification of the dopaminergic phenotype also by modulating *Pitx3*. These findings are not in contrast with the hypothesis that Nurr1 could interact with Pitx3 at the protein level either directly or through a third partner, to regulate common target genes, as has been suggested by others [22], [23]. Thus *Pitx3* regulation could take place at various levels, and at least in part, appears to be regulated by Nurr1 in mDA neurons.

In synthesis, our report sheds new light on the role of Nurr1 in mDA neuron differentiation and maintenance, positioning this TF at a high hierarchical level in the regulation of this neuronal phenotype.

## Methods

### Cell lines

The MN9D-Nurr1^Tet On^ cell line was kindly provided by Thomas Perlmann [13]. Cells were maintained at 37°C, with 5% CO_2_ in DMEM/F12 medium (Life Technologies, Milan, Italy) supplemented with 10% FBS (Euroclone, Milan, Italy), 100 U/ml penicillin and 100 µg/ml streptomycin (Sigma, Milan, Italy). Cells were grown in poly-D-lysine (Sigma) coated flasks as previously described. *Nurr1* expression was induced by addition of 3 µg/ml doxycycline (Sigma) to the culture medium or with 3×FLAG-Nurr1 transfection. Cells were grown as above except that the 10% serum was changed in B27 supplement (Life Technologies). *Nurr1* expression was induced by addition of 3 µg/ml doxycycline (Sigma). HeLa cells (ATCC, LGC Standards, Italy) and MN9D [24] were used as well.

### Plasmids construction

*Pitx3* promoter (pPitx3) sequence, analyzed using MatInspector Release professional 8.0 from Genomatix software package (GmbH, Munich, Germany, http://www.genomatix.de/), was amplified and cloned in pGL3-basic firefly luciferase reporter vector (Promega, Milan, Italy). The plasmid pGL3-pPitx3 sequence was confirmed by sequencing. Mutant constructs of pPitx3 were generated with the Quick-change site-direct mutagenesis kit (Stratagene), according to the manufacturer's protocol.

Flag-tagged full-length (FL) mouse *Nurr1*, cloned into 3×FLAG vector (Sigma), was kindly provided by Kaoru Saijo [11]. Full-length (FL) of human *RXRα* was kindly provided by Philip Lefevbre [25].

### Transient transfection and luciferase assay

700 bps region upstream the 5′UTR of the *Pitx3* promoter was cloned into a pGL3 basic vector (pPitx3-luc) and co-transfected with the 3×FLAG-Nurr1 plasmid into HeLa cells using lipofectamine 2000, according to the manufacturer's protocol. *Renilla* luciferase vector, carrying the Simian vacuolating virus 40 promoter (pRL-SV40), were used as an internal control. For the dual (firefly and *Renilla*) luciferase assays, cells were extracted with passive lysis buffer (Promega), and 10 µl of cell extract was used according to the manufacturer's protocol. Values were expressed as a ratio of luminescence signals between the luciferase reporter and the *Renilla*. The data were performed in triplicate.

### RNA isolation and Real time PCR

Total RNA was isolated using Tri-Reagent (Sigma, Milan, Italy) according to the manufacturer's instructions. The analyses were always carried out in triplicate samples for each experimental point analyzed and were processed separately. The yield and integrity of RNA were determined by spectrophotometric measurement of A_260_ and agarose gel electrophoresis respectively. Briefly, 2 µg of RNA were reverse transcribed, using random hexanucleotides (New England Biolabs Inc., Milan, Italy, 6 mM) and 200 U of Moloney-murine leukemia virus reverse transcriptase (Ambion). Gene specific primer sets (Table 1) used for quantitative real time PCR (qRT-PCR, Applied Biosystem, Milan, Italy) were designed using OLIGO 6 software according to manufacturer's instructions, in order to obtain amplified fragments with comparable length (around 100 bp). SYBR Green qRT-PCR reactions were performed in 96-well plates using 7900HT Fast *Real*-*Time* PCR System (Applied Biosystem). Thermal cycling conditions comprised initial steps at 50°C for 2 minutes and 95°C for 10 minutes, followed by 40 cycles at 95°C for 15 seconds and 60°C for 1 minute. All samples were run in triplicate. Amplification efficiency of each primer pair was verified by performing qRT-PCR using different template dilutions. Gene expression levels were quantified from real-time PCR data by the comparative threshold cycle (*CT*) method [26] using hypoxanthine phosphoribosyl transferase (HPRT) as an internal control gene. The fractional number of PCR cycles *CT* required to obtain a given amount of qRT-PCR product in the exponential phase of amplification was determined for the gene of interest and for HPRT in each RNA sample. The relative expression level of the gene of interest was then expressed as 2^−ΔΔ*CT*^ where Δ*CT* = *CT* gene of interest - *CT* HPRT.

**Table 1:** Primers (5′-3′) used for Real time-PCR.

Gene of interest	Primers Sequence
**Hprt**	F: TGGGAGGCCATCACATTGTR: AATCCAGCAGGTCAGCAAAGA
**Nurr1**	F: CAACTACAGCACAGGCTACGAR: GCATCTGAATGTCTTCTACCTTAATG
**Pitx3**	F: GACACTGGCCGCCCAAGGR: AGGCCCCACGTTGACCGA
**TH**	F: CCTTTGACCCAGACACAGCAR: ATACGAGAGGCATAGTTCCTGAG
**Vmat2**	F: TTGCTCATCTGTGGCTGGGR: TGGCGTTACCCCTCTCTTCAT

The table shows the forward (F) and reverse (R) primers used in Real time PCR for the following genes: hypoxanthine-phosphoribosyl-transferase (*Hprt*), *Nurr1*, the paired-like homeodomain transcription factor 3 (*Pitx3*), tyrosine hydroxylase (*TH*), vesicular monoamine transporter 2 (*Vmat2*).

### Western Blotting

For Western blot analyses three different cultures samples were lysed in RIPA Buffer in presence of protease inhibitors (Roche, Milan, Italy). 50 µg/lane of proteins were separated on 12% SDS-polyacrilamide gel and transferred to PVDF membranes (Amersham, Milan, Italy). Filters were probed for 2 hrs at room temperature or overnight at 4°C with the following antibodies: anti-Nurr1/Nur77 (E-20 sc-990 X, Santa Cruz Biotechnology Inc., Milan, Italy, 1: 5000), anti-ß-actin (Sigma, 1∶1000), anti-Pitx3 (Abcam, Cambridge, UK 1∶250), anti-Neuronal Class III ß-Tubulin (TUJ1, Convance, Milan, Italy, 1∶1000). After washing, immunoblots were incubated with goat anti-rabbit (Bio-Rad, 1∶1500) or anti-mouse IgG antibodies (Amersham, 1∶5000) and the reaction detected with the ECL plus procedure (Amersham). The relative protein levels were determined by densitometry and compared with the protein level of ß-actin and TUJ1 either in control or in treated cells.

### Chromatin Immunoprecipitation (ChIP)

5×10^6^ MN9D cells were transfected with 3×FLAG-Nurr1 using lipofectamine 2000, fixed 24 hrs post transfection with 1% formaldehyde for 15 min at r/t and the reaction stopped by addition of 125 mM glycine for 5 min. The pellets, washed in cold PBS with proteinase inhibitors, were lysed in 5 ml of Lysis Buffer [5 mM Pipes pH 8.0, 85 mM KCl, 0.5% NP40 and proteinase inhibitors]. After the centrifugation at 2000 rpm for 5 min, nuclei were dissolved in High Salt Lysis Buffer [HSLB- 1× PBS, 1% NP-40, 0.5% Sodium Deoxycholate, 0.1% SDS and Protease Inhibitor Cocktail]. Chromatin was fragmented by sonication in DNA fragments from 200–1000 bp, cells debris removed by centrifugation and samples pre-cleared with 50 µl Protein A/G Plus-Agarose (Santa Cruz Biotechnology, sc-2003) for 2 hrs at 4°C. Immunoprecipitations of cross-linked complexes were performed by overnight incubation at 4°C using 1 µg of goat anti- Nurr1/Nur77 antibody (E-20 sc-990 X, Santa Cruz Biotechnology) or 1.5 µg of anti-mouse 3×FLAG antibody (Sigma). For each experiment, a sample without antibody was carried out in parallel as a control for nonspecific background. Protein A/G Plus-Agarose (50 µl) were added, incubated at 4°C for 2 hrs, spun at 2000 rpm, washed twice in HSLB, four times in Wash Buffer [100 mM Tris (pH 8.0), 500 mM LiCl, 1% NP-40 and 1% Deoxycholate]. The supernatant of bound and unbound samples was then incubated at 67°C in 100 mM NaHCO_3_ and 1% SDS to elute immune complexes. DNA was phenol-chloroform extracted, ethanol precipitated, UV quantified, and used for PCR or Real Time with primers spanning the NBRE-like sites in pGL3-pPitx3.

### Statistical analysis

The analyses data have been described above. For all other experiments, analysis of variance was carried out, followed by post hoc comparison (ANOVA, Scheffè F-test). Data were expressed as mean +/− SEM.
