# Correction: Direct Regulation of Pitx3 Expression by Nurr1 in Culture and in Developing Mouse Midbrain

**DOI:** 10.1371/journal.pone.0233918

**Published:** 2020-05-22

**Authors:** Floriana Volpicelli, Roberto De Gregorio, Salvatore Pulcrano, Carla Perrone-Capano, Umberto di Porzio, Gian Carlo Bellenchi

After publication of this article [1], questions were raised about some of the reported results:

- In Figure 2A there appears to be a discontinuity in background after lane 1 of the β-actin blot, when brightness and contrast are adjusted. The discontinuity is also visible in the original underlying image for this blot (S1 File), which matches the image in the published figure. The original Pitx and TUJ1 blot images supporting Figure 2A are in S2 and S3 Files, respectively. Lanes 1–6 and 8–13 in each of these image files show data from the same blot and exposure. Quantitative data supporting the graph in Figure 2A are in S4 File, and the results of quantification using TUJ1 control data are in S5 File.
- In Fig 4C, there is a vertical discontinuity between lanes 1, 2 in the pPitx3 panel. The authors noted that an extra control lane from the original image was spliced out when preparing the figure and apologized for not having indicated the splicing clearly in the published figure and its legend. An updated Fig 4 is provided here in which this has been addressed. The original image data underlying the pPitx3 results in Fig 4C are in S6 File. Fig 4C shows a representative result from experiments using 0.5 μg antibody per ChIP, and the overall results for ChIP-Real time PCR experiments using 2 μg antibody per ChIP are summarized quantitatively in Fig 4D. Quantitative data supporting pPitx3 results in Fig 4D are in S7 File, and replication data for this experiment are in S8 File. Raw quantification data for the BDNF experiment represented in Fig 4D are no longer available. The following issues arose in our editorial review of the data files provided in support of Fig 4C and 4D:
  - Among the replicate data (S6 File), the data shown in Fig 4C show the weakest band for IgG and the strongest band for Nurr. As such, the data observed across experimental replicates provides weaker support for the difference between IgG and Nurr1 compared to images in Fig 4C. The quantitative data in Fig 4D show the cumulative results from multiple experimental replicates.
  - During the editorial assessment of the quantitative data, questions were raised about the different results shown in S7 File for experiments done using 0.5, 1, 2, or 4 μg of input RNA: unlike the 0.5 and 2 μg conditions (2 μg results are reported in the article), the 1 and 4 μg data do not appear to support enrichment over background. The authors commented that they did not repeat the 4 μg experiments for which they estimated there were saturating amounts of antibody that may have impacted background. The authors also confirmed that the 1 μg antibody immunoprecipitation was less efficient compared to the 0.5 and 2 μg experiments, and they noted that they included only the 0.5 and 2 μg conditions in replicate experiments.

The corresponding author stated that original data underlying all other results reported in the article are available upon request.

**Fig 4:**
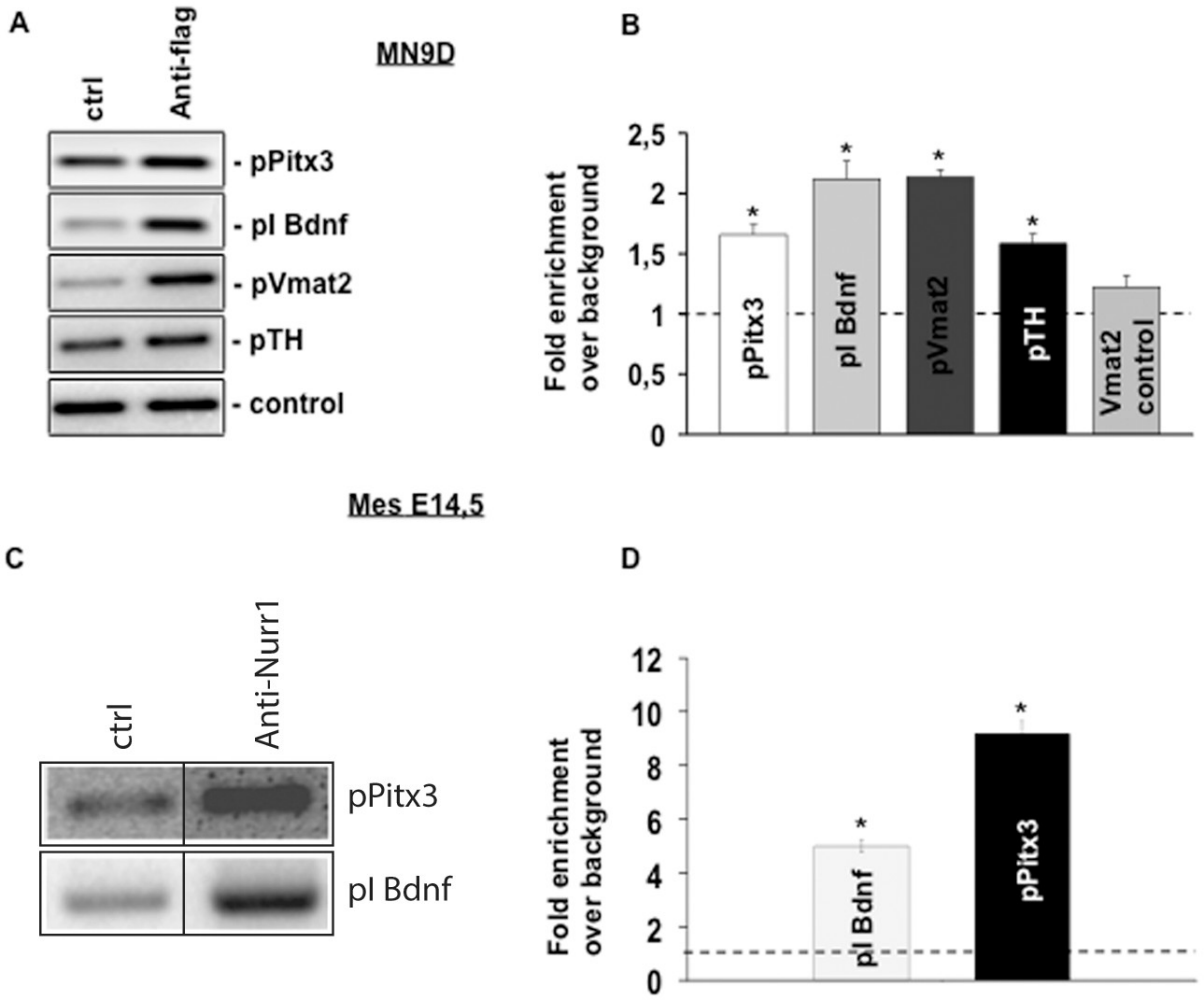
Nurr1 directly regulates *Pitx3* expression by binding to its promoter. (A) ChIP-PCR analysis performed in MN9D transfected with 3×FLAG-Nurr1 and immunoprecipitated with anti-FLAG antibody shows a significant enrichment of *Pitx3*, *Bdnf* and *Vmat2* promoter regions. No enrichment was observed when an unrelated region of the *Vmat2* promoter was used [19]. The inserts show representative PCR amplified fragments after ChIP. (B) ChIP-Real time PCR in MN9D transfected with 3×FLAG-Nurr1 and immunoprecipitated with anti-FLAG antibody. The diagram shows the fold enrichment over background (dotted line) for *Pitx3*, *Bdnf* and *Vmat2* promoter regions. (C) ChIP-PCR validation performed in E14.5 midbrain and immunoprecipitated with Nurr1 antibody shows a significant enrichment of the *Pitx3* and *Bdnf* promoter regions. A representative PCR amplified fragment is shown into the insert. The vertical black line in the pPitx3 panel indicates an image splice junction: the panel shows data obtained using the same original gel and exposure, additional lanes were removed when preparing the figure. The original gel image is in S6 File. (D) The diagram shows the ChIP-Real time PCR quantitation of *Bdnf* and *Pitx3* promoter region in E14.5 midbrain and immunoprecipitated with Nurr1 antibody. Results are expressed as mean ± SE of at least three independent experiments. Asterisks (*) represent p≤0.01 when compared to control (ANOVA, Scheffè F-test).

## Supporting information

S1 File Original image underlying β-actin blot in Figure 2. (JPG)

S2 File Original image underlying Pitx3 blot in Figure 2. (JPG)

S3 File Original image underlying β-tubulin blot in Figure 2. (JPG)

S4 File Quantitative data underlying graph in Figure 2A. (XLSX)

S5 File Quantification of Figure 2 western blot data, normalized to TUJ1 results. (XLSX)

S6 File Original gel image underlying the pPitx3 result reported in Fig 4C. The experiment included two concentrations of antibodies for both IgG and Nurr1. Samples were loaded as follows: Lane 1: 0.5 μg IgG; Lane 2: 0.5 μg IgG; Lane 3: 2 μg IgG; Lane 4: 2 μg IgG; Lane 5: 0.5 μg Nurr1, Lane 6: 0.5 μg Nurr1; Lane 7: 2 μg Nurr1; Lane 8: 2 μg Nurr1.
(PDF)

S7 File Results from one representative ChIP experiment, which included four antibody concentrations, including two (1 μg, 4 μg) that were not included in subsequent experimental replicates. For the experiment represented in this file, there was one experimental sample per condition; cells F27-S42 in the Excel file include data for the pPitx3-I experiment, the 2 μg results from this experiment represent one replicate of the three independent experiments summarized in the Fig 4D graph. The raw data for the other replicates are not available. Two potential binding sites for Nurr1 on the Pitx3 promoter were identified, named pPitx3-I and pPitx3-II. Only promoter I (pPitx3-I) is significantly enriched after the ChIP and was assessed in following experiments. Quantification of Pitx3 promoter by qPCR after ChIP. Data obtained with 2 μg antibody are reported in the original Fig 4D. The efficiency of IP was calculated as follows: (a) ΔC_t_ (Ct_IP_ − Ct_INPUT_) was calculated considering the abundance of a target DNA sequence (bound or immunoprecipitated, Ct_IP_) relative to input chromatin (Ct_INPUT_); (b) the results were expressed as 2^-ΔCt^ or as 2^-ΔCt^ x 100 (.xls file column O).
(XLSX)

S8 File Results from replication of the Fig 4D experiment in which Real Time PCR analysis was performed on the Pitx3 promoter after ChIP. (XLS)
