# Supplementary figures and images for: Correction: Direct Regulation of Pitx3 Expression by Nurr1 in Culture and in Developing Mouse Midbrain
Source: PLoS One. 2020 May 22;15(5):e0233918. doi: 10.1371/journal.pone.0233918 (PMC7244126; doi:10.1371/journal.pone.0233918)

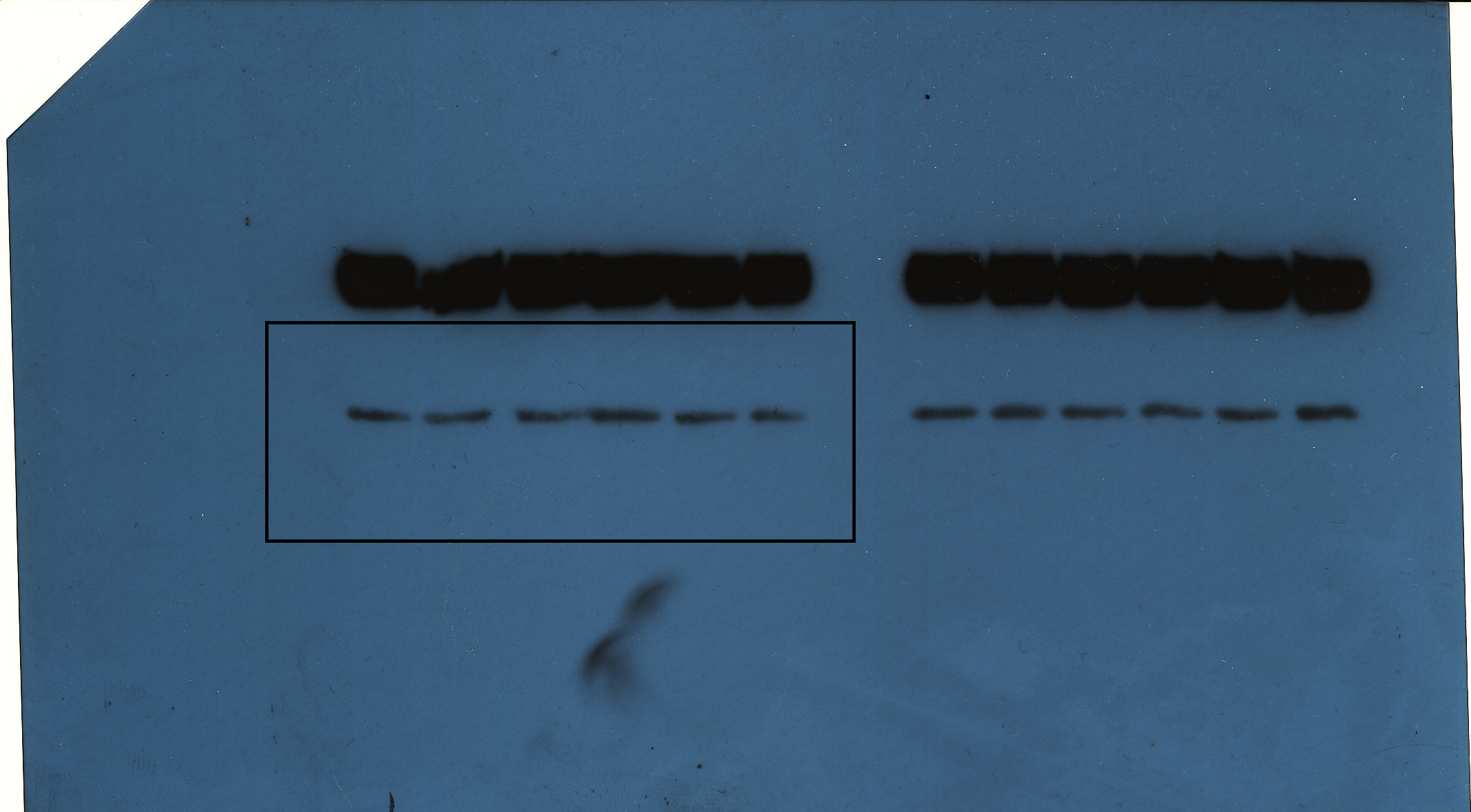

Supplement: S1 File — (JPG) [file pone.0233918.s001.jpg]

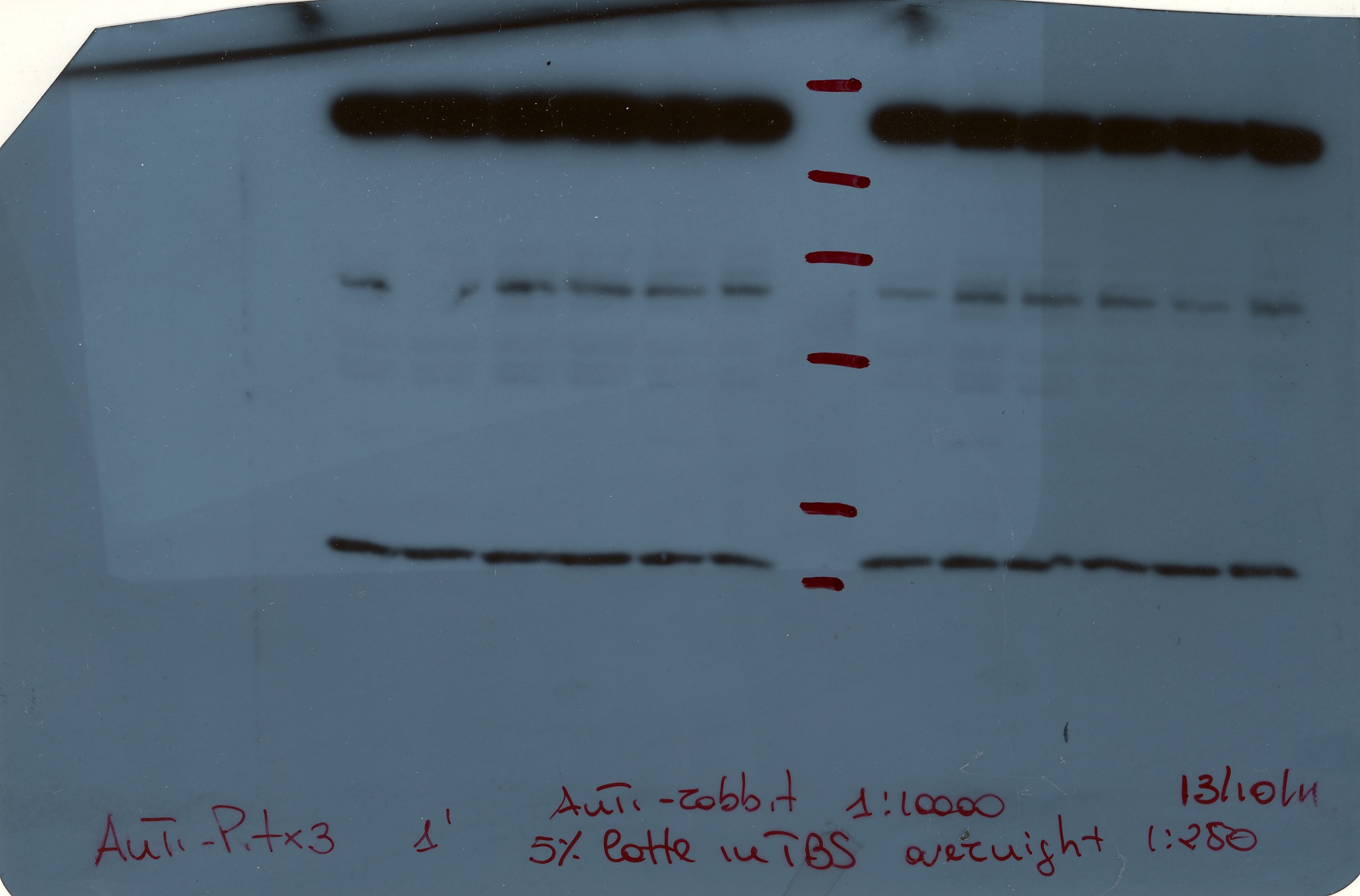

Supplement: S2 File — (JPG) [file pone.0233918.s002.jpg]

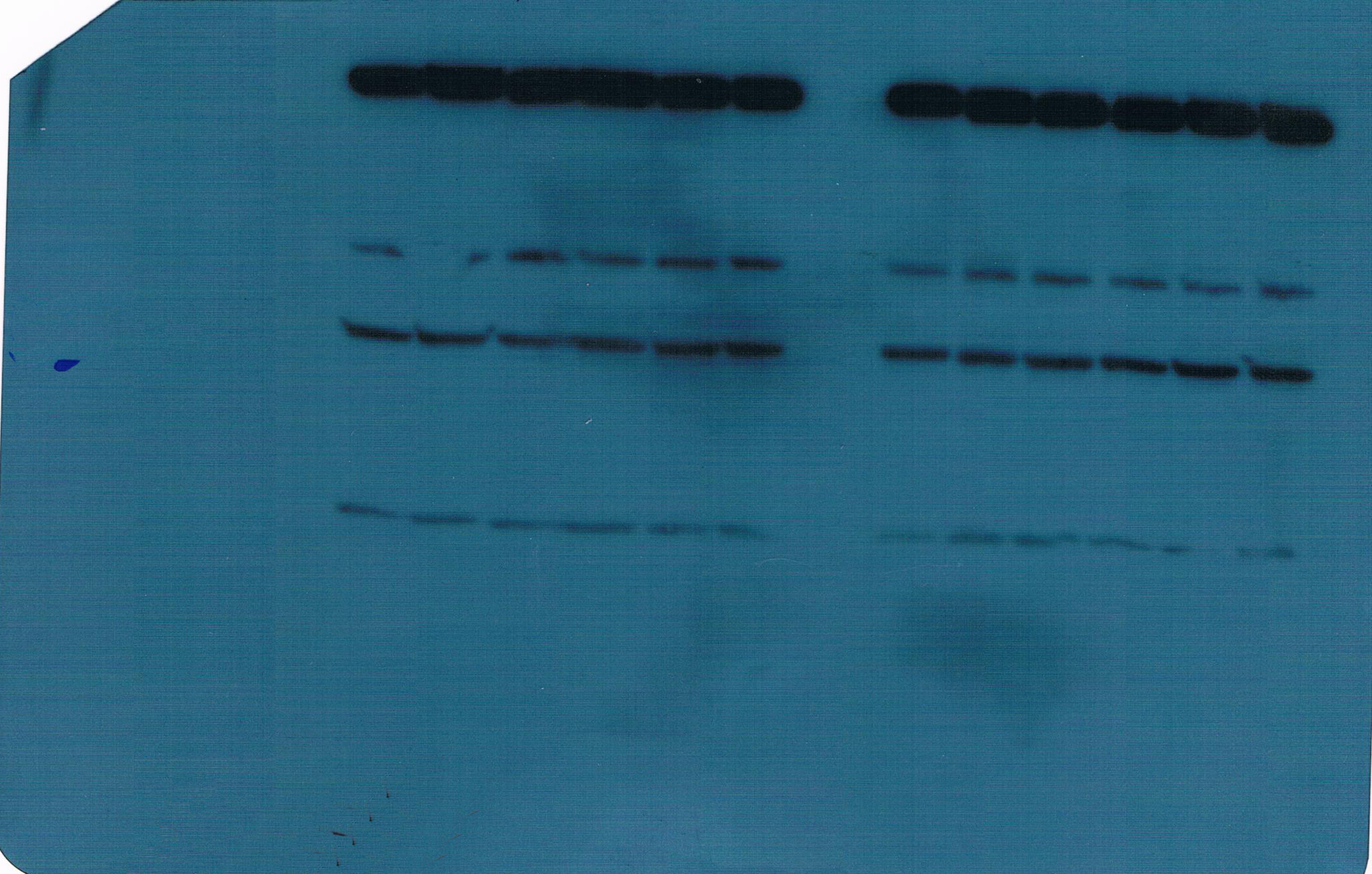

Supplement: S3 File — (JPG) [file pone.0233918.s003.jpg]

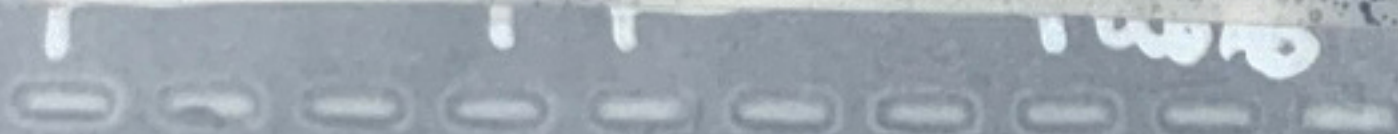

lgG

Anti

up  
Ab

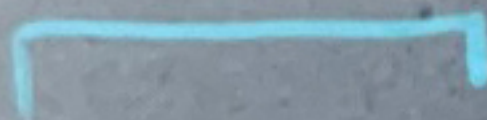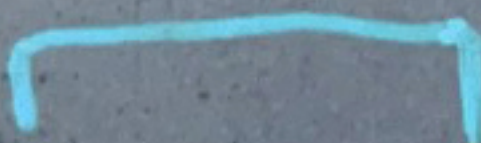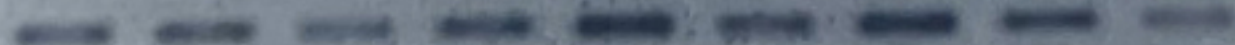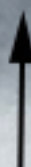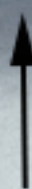

30/03/2009

297x3 I

Supplement: S6 File — The experiment included two concentrations of antibodies for both IgG and Nurr1. Samples were loaded as follows: Lane 1: 0.5 μg IgG; Lane 2: 0.5 μg IgG; Lane 3: 2 μg IgG; Lane 4: 2 μg IgG; Lane 5: 0.5 μg Nurr1, Lane 6: 0.5 μg Nurr1; Lane 7: 2 μg Nurr1; Lane 8: 2 μg Nurr1. (PDF) [file pone.0233918.s006.pdf]
